# Genomics assisted functional characterization of *Bacillus velezensis* E as a biocontrol and growth promoting bacterium for lily

**DOI:** 10.3389/fmicb.2022.976918

**Published:** 2022-11-30

**Authors:** Bingyu Li, Junyi Wan, Jingjing Sha, Mengyuan Tian, Mengchen Wang, Xinyuan Zhang, Wei Sun, Yanrong Mao, Jie Min, Yiyuan Qin, Yuqing Liu, Wenhe Wang, Xiangfeng He

**Affiliations:** ^1^Beijing Advanced Innovation Center for Tree Breeding by Molecular Design, Beijing University of Agriculture, Beijing, China; ^2^College of Landscape Architecture, Beijing University of Agriculture, Beijing, China; ^3^Beijing Laboratory of Urban and Rural Ecological Environment, Beijing, China

**Keywords:** lily basal rot, lily gray mold, *Bacillus velezensis*, genome analysis, plant growth promotion, biocontrol

## Abstract

Lily (*Lilium* spp.) is one of the most famous ornamental flowers globally. Lily basal rot (also known as root rot or stem rot) and lily gray mold have seriously affected the yield and quality of lily, resulting in huge economic losses. In this study, bacterial strain E was isolated from a continuous lily cropping field. Strain E displayed high control efficiency against lily basal rot and gray mold, caused by *Fusarium oxysporum* and *Botrytis cinerea* respectively, and promoted the occurrence of scale bulblets. Strain E displayed strong inhibitory effects against several other plant pathogenic fungi and two pathogenic bacteria in dual culture and disc diffusion assays, respectively. Whole genome sequencing revealed that strain E contained a 3,929,247 bp circular chromosome with 4,056 protein-coding genes and an average GC content of 47.32%. Strain E was classified as *Bacillus velezensis* using genome-based phylogenetic analysis and average nucleotide identity and digital DNA–DNA hybridization analyses. A total of 86 genes and 13 secondary metabolite biosynthetic gene clusters involved in antifungal and antibacterial activity, plant growth promotion, colonization, nutrient uptake and availability were identified in the genome of strain E. *In vitro* biochemical assays showed that strain E produced siderophores, proteases, cellulases, biofilms, antifungal and antibacterial substances, and exhibited organic phosphate solubilization and swimming and swarming motility, which were consistent with the results of the genome analysis. Colonization analysis showed that strain E could colonize the root of the lily, but not the leaf. Overall, these results demonstrate that *B. velezensis* strain E can be used as a potential biofertilizer and biocontrol agent for lily production.

## Introduction

Lily (*Lilium* spp.) is a perennial bulbous flower belonging to the Liliaceae family. Owing to its large and colorful flowers, lily is one of the most famous ornamental flowers worldwide. China is one of the world’s largest producers of lily cut flowers. In recent years, diseases affecting lily have become increasingly serious because of continuous cropping. Lily basal rot (also known as root rot or stem rot) and lily gray mold have seriously affected the yield and quality of lily, and resulting in huge economic losses (Cui et al., 2018; He et al., 2019). Lily basal rot is a soil-borne disease that mainly affects the underground parts of plants and results in wilting, yellowing, and rotting of the bulbs. *Fusarium oxysporum* f. sp. lilii is the main pathogen that causes lily basal rot (Straathof and Löffler, 1994). Gray mold, caused by the fungus *Botrytis cinerea*, is a common fungal disease in flowers and one of the most serious diseases in *Lilium* (de Cáceres et al., 2015). Lily flowers infected by *B. cinerea* display white, brown, or light brown hygrophanous lesions, and even decay severely (Ma et al., 2018). Currently, chemical control methods are employed to control lily basal rot and lily gray mold. However, chemical control has shown dwindling effects because the pathogens are seed-borne or soil-borne, and pathogens develop resistance to the chemicals used (Harold et al., 1997; He et al., 2019). Simultaneously, the long-term, large-scale and repeated use of chemical pesticides has caused environmental pollution and safety problems in edible and medicinal lily. Therefore, pollution-free biological control is the preferred choice for controlling lily diseases.

Biocontrol has become a promising strategy for controlling various plant diseases (Zhang et al., 2017; Fan et al., 2018; Mao et al., 2020; Ding et al., 2021). To date, several biological control agents (BCAs) have been reported. However, only a limited number of strains can be commercialized, because storing and transporting BCAs as stable products is a difficult process (Fan et al., 2018; Ding et al., 2021). The Gram-positive *Bacillus* is one of the most important commercial BCAs because it can form long-lived, heat- and desiccation-resistant endospores (de Benito Armas et al., 2008; Fan et al., 2018). Some strains of *Bacillus* exhibit biological activity against plant pathogens, including bacteria, fungi, and nematodes, by secreting extracellular metabolites and enzymes (Rabbee et al., 2019; Miljakovi et al., 2020). Additionally, *Bacillus* strains can trigger induced systemic resistance (ISR) in plants to protect them from attacks by various plant pathogens (Fan et al., 2018; Miljakovi et al., 2020). In addition to being biocontrol agents, *Bacillus* strains can promote plant growth through diverse mechanisms, such as the production of 1-Aminocyclopropane-1-caroxylate deaminase (ACC), siderophores and phytohormones, and processes such as phosphate solubilization and nitrogen fixation (Bhattacharyya and Jha, 2012; Fan et al., 2018).

*Bacillus velezensis* is one of the most valuable BCAs in the genus *Bacillus*. *B. velezensis* was first isolated from environmental samples in 2005 (Ruiz-García et al., 2005). Based on the results of recent phylogenetic analyses, several *Bacillus* species, including all strains previously classified as *B. velezensis*, *B. methylotrophicus*, and *B. amyloliquefaciens* subsp. *plantarum*, have been reclassified as *B. velezensis* (Dunlap et al., 2016; Rabbee et al., 2019).

So far, many strains of *B. velezensis* have been reported to promote plant growth and control diseases in various plants, including shallot, soybean, lotus, walnut, *Quercus*, Japanese cypress, strawberry, sesame, apple, ginger, pepper, poplar, tomato, grape, tobacco, watermelon, wheat, maize, and cotton (Cai et al., 2017; Jiang et al., 2019; Aulia Rahma et al., 2020; Bayisa, 2020; Wang et al., 2020; Choub et al., 2021; Ding et al., 2021; Hamaoka et al., 2021; Han et al., 2021; Liang et al., 2021; Liu et al., 2021; Masmoudi et al., 2021; Moon et al., 2021; Sachin et al., 2021; Shin et al., 2021; Won et al., 2021; Adeniji and Babalola, 2022; Hong et al., 2022; Yuan et al., 2022). Although *B. velezensis* has attracted increasing attention as a beneficial microorganism, its application in the control of ornamental flower diseases has not yet been reported.

In this study, antagonistic bacteria against a wide range of fungal pathogens, including *F. oxysporum* and *B. cinerea*, were isolated from the soil. Among these, *B. velezensis* E had the widest spectrum of antifungal activity and exhibited the best control on lily basal rot and gray mold. *B. velezensis* E promoted plant growth and produced active substance, as was detected using plant growth and biochemical assays. Whole-genome analysis of strain E revealed the presence of genes involved in pathogens biocontrol and plant growth promotion. Overall, this research showed that *B. velezensis* E can potentially be used to control lily basal rot and gray mold and promote lily bulblet morphogenesis in scale propagation.

## Materials and methods

### Isolation and purification of bacteria from soil samples

Soil samples for bacterial isolation were obtained from approximately 5 cm below the soil surface in the continuous lily cropping field of Yanqing District, Beijing, China. Five grams of soil sample were suspended in 50 ml of sterile water and were incubated for 30 min at 28°C with shaking (150 rpm). After 10-fold serial dilutions, 0.5 ml of suspension was placed on Luria-Bertani (LB) plate and incubated at 28°C. Bacteria from the single colonies were further purified by streaking onto LB agar plates. Bacterial isolates were inoculated in LB broth medium and incubated overnight at 28°C and 180 rpm. After incubation, the isolates were mixed with an equal volume of 40% glycerol and stored at –80°C for further experimentation.

### Antagonism assay against phytopathogenic fungi and bacteria

Bacterial isolates were assessed for antifungal activities against *Fusarium oxysporum*, *Fusarium equiseti*, *Botrytis cinerea*, *Rizoctonia solani*, *Alternaria solani*, and *Phytophthora capsica* using dual culture assay. A mycelium plug (0.5 cm diameter) of actively growing fungi was placed at the center of a Potato Dextrose Agar (PDA) plate. The suspension of bacterial isolates (OD600 = 1, 5 μl for each point) was then inoculated onto the PDA plate at three points at a distance of 2 cm from the mycelium plug, followed by growth at 28°C in the dark. Antifungal activity was evaluated using Singh’s method (Singh et al., 2013) until the mycelia of fungal pathogens grew to the edge of the Petri dish in the control. Three replicates were used for each treatment.

The antagonistic activities of the bacterial isolates against *Xanthomonas arboricola* and *Pantoea agglomerans* were determined using a disc diffusion assay with minor modifications (Durairaj et al., 2018). A total of 200 μl of overnight pathogenic bacterial culture grown in LB broth was evenly coated on the surface of the LB solid medium. Then, 5 μl of the fermentation suspension of bacterial isolates (OD600 = 1) was added to sterile filter paper with a diameter of 6 mm and placed on the culture medium with pathogenic bacteria. Sterile water was used as a negative control.

### Inhibitory effect of bacterial culture filtrate (BCF) on spore germination, germ tube elongation, and mycelial growth of fungal pathogens

To prepare spore suspensions of fungal pathogens, 0.5 cm mycelial plugs of *B. cinerea* and *F. oxysporum* were placed on PDA medium and incubated at 28°C for 10 days. The fully sporulated fungal plate was washed with sterile distilled water containing 0.03% Tween 80 and gently scratched with sterile spatula. The spore suspension was filtered using sterile gauze and adjusted to 1 × 10^5^ spores/ml by diluting with Potato Dextrose Broth (PDB).

To prepare BCF, a single colony of *B. velezensis* E was pre-inoculated in LB broth medium and incubated overnight at 28°C in a shaking incubator set at 180 rpm. Then, 100 μl of broth culture was inoculated into LB broth medium (100 ml) and incubated at 28°C and 180 rpm for 2 days. The bacterial suspension was then centrifuged at 12,000 rpm for 10 min. The bacterial supernatant was passed through 0.22 μm pore-size filter membranes to obtain a BCF without bacterial cells.

To estimate the inhibitory effect of BCF on fungal pathogens, the obtained BCF was added to the spore suspension of the fungal pathogens to obtain final concentration of 10% BCF. LB broth was added to the spore suspension of the fungal pathogen as a control. A mixture of fungal spores and BCF was incubated at 28°C and 180 rpm. Germination of 100 spores and germ tube lengths of 20 spores from each treatment were observed and recorded after 10 h. Morphological changes in the fungal hyphae were observed and photographed after incubation for 3 days. The fungal hyphae were harvested using Whatman No.1 filter paper after the mixture of fungal spores and BCF was incubated for 2 days and then dried at 60°C for 2 days, followed by the measurement of dry weight of hyphae.

### Identification of bacterial isolate by 16S rDNA sequence analysis

Genomic DNA was extracted from the isolate using the Bacteria Genomic DNA Extraction Kit (Tiangen Corporation Ltd., Beijing, China) according to the manufacturer’s instructions. For 16S rRNA gene amplification, genomic DNA was amplified using the universal bacterial primers F27 (5′-AGAGTTTGATCCTGGCTGGCTCAG-3′) and R1492 (5′ -TACGGCTACCTTGTTACGACTT-3′). The PCR mix contained 2 μl of DNA template (100 ng/μl), 2 μl each of F27 and R1492 (10 μm), 25 μl of 2 × Taq PCR Green Mix (Ding-guo Changsheng Corporation Ltd., Beijing, China), and topped to a final volume of 50 μl using RNase-free water. The PCR reaction mix was incubated at 94°C for 5 min, and then 94°C for 1 min, 55°C for 1 min, 72°C for 2 min for 30 cycles, followed by a final extension at 72°C for 10 min. The PCR product was verified by agarose gel electrophoresis and purified using a TIANgel Midi Purification Kit (Tiangen Corporation Ltd., Beijing, China) according to the manufacturer’s instructions. The purified product was cloned into the pTOPO-TA simple vector (Aidlab Corporation Ltd., Beijing, China) and sequenced at BGI (Shenzhen, China). The obtained sequence (about 1,500 bp) was aligned to the GenBank database using NCBI BLAST.

### Evaluation of biocontrol activities and plant growth-promoting traits of bacterial strain *in vitro*

Siderophore production: The Chrome Azurol S (CAS) assay was used to detect siderophore production (Pérez-Miranda et al., 2007). All glassware used for the detection of siderophores was washed with 6 M HCl (Esmaeel et al., 2016). Ten microliters of freshly grown bacterial culture was inoculated on CAS agar plates, followed by incubation at 28°C for 4 days. The formation of a yellow-orange halo around the bacterial colony indicated siderophore production. Fresh LB broth without bacteria was used as a negative control.

Phosphate solubilization: Inorganic phosphorus (IP medium) and organic phosphorus solid medium (OP medium) were used to determine inorganic and organic phosphate solubilization, respectively (Teng et al., 2019). Ten microliters of freshly grown bacterial culture was inoculated on IP or OP medium, followed by incubation at 28°C for 4 days. The formation of a halo zone around the bacterial colony in IP or OP medium indicated inorganic or organic phosphate solubilization.

Proteolytic activity: Skim milk agar plates were used to test the ability of antagonistic bacteria to produce protease. This experiment was performed using a previous described procedure with minor modifications (Adhikari et al., 2017). The bacteria were dipped from freshly grown single colonies with sterile toothpicks and inoculated onto skim milk agar plates, followed by incubation at 28°C for 2 days. The development of a clear halo around the bacterial colony indicated protease production.

Cellulase production: Carboxymethyl cellulose (CMC) agar was used to determine the ability of antagonistic bacteria to produce cellulase; this experiment was performed using a previously described method with minor modifications (Syed-Ab-Rahman et al., 2018). The bacteria were dipped from freshly grown single colonies with sterile toothpicks and inoculated onto CMC agar plates, followed by incubation at 28°C for 4 days. The Congo red solution (1 g/l) was poured onto the surface of the plate for 60 min. Finally, unbound Congo red was removed by rinsing the plate with NaCl solution (1 M). The formation of a clear halo around the bacterial colony confirmed cellulase production.

Motility assay: The swarming and swimming motility of antagonistic bacteria were analyzed using previously reported methods (Ding et al., 2021; Thomloudi et al., 2021). A total of 10 ml of bacteria were grown to mid-log phase at 28°C in LB broth, centrifuged at 4,000 rpm, and resuspended in 100 μl of PBS. Subsequently, a 5 μl drop of bacterial suspension was inoculated at the center of freshly prepared LB plates containing 0.7 or 0.3% agar for swarming and swimming assays. After incubation at 30°C for 8 h, the appearance of a bacterial zone was observed.

Biofilm production: The ability of antagonistic bacteria to form biofilms was determined according to Rondeau’s method (Rondeau et al., 2019) with slight modifications. The antagonistic bacteria were inoculated into 5 ml of LB broth and incubated overnight at 28°C. The bacterial culture was diluted to an OD 600 of 0.2 with LB broth and added to a sterile polypropylene tube (300 μl per tube). After incubation at 28°C for 1 day without agitation, the medium was removed and washed with distilled water thrice. Subsequently, 600 μl of 1% (*w*/*v*) crystal violet was added to each tube and incubated at room temperature for 30 min. The crystal violet was removed from the tube by rigorous washing with distilled water thrice. Following this, 1.2 ml of 100% methanol was added to each tube. After incubation for 30 min, the biofilm formation was quantified by measuring the absorbance at 540 nm. Five replicates were used for each treatment.

### Genome sequencing, assembly, annotation, and analysis

The genomic DNA of strain E was extracted using the cetyltrimethylammonium bromide (CTAB) method (Wilson, 2001). The concentration of genomic DNA was determined using a Qubit fluorometer (Invitrogen, United States). Sample integrity and purity were detected using electrophoresis on 1% agarose gel and NanoDrop 2000 Spectrophotometer (Thermo Scientific, United States). The genome of strain E was sequenced using the PacBio sequel II and DNBSEQ platforms at the Beijing Genomics Institute (BGI, Shenzhen, China). After removing low-quality raw reads using SOAPnuke (v1.5.6; Chen et al., 2018), high-quality reads were assembled into a corrected circular consensus sequence using Canu (v1.5; Koren et al., 2017), Proovread (Hackl et al., 2014), and GATK.^1^ Gene prediction was performed on the assembled genome sequence of strain E using glimmer3^2^ with Hidden Markov. tRNAscan-SE (Lowe and Eddy, 1997), RNAmmer (Lagesen et al., 2007), and the Rfam database^3^ were used to identify tRNA, rRNA, and sRNAs. Tandem repeat annotation was performed using the Tandem Repeat Finder,^4^ and minisatellite DNA and microsatellite DNA were selected based on the number and length of repeat units. Functional annotation was accomplished by analysis of protein sequences. To obtain their corresponding annotations, all genes from strain E were aligned with different databases, including the Kyoto Encyclopedia of Genes and Genomes (KEGG), Clusters of Orthologous Groups (COG), Non-Redundant Protein Database databases (NR), Swiss-Prot, Gene Ontology (GO), and InterPro using Diamond software.

The phylogenetic identity of strain E was established by calculating digital DNA–DNA hybridization (dDDH) using the genome-to-genome distance calculator website service (GGDC 2.1; Meier-Kolthoff et al., 2013)^5^ and orthologous average nucleotide identity (OrthoANI) using the web service EzBioCloud (Yoon et al., 2017).^6^ The OrthoANI values 95–96% and dDDH values 70% were considered as species delineation threshold values (Meier-Kolthoff et al., 2013; Yoon et al., 2017). Phylogenomic analysis of strain E was carried out using the Type (strain) Genome Server^7^ and the tree was generated using FastME 2.1.6.1 (Lefort et al., 2015) from GBDP distances calculated from genome sequences.

Biosynthetic gene clusters (BGCs) of potential antimicrobial compounds were identified by antiSMASH 6.0^8^ and BAGEL 4 (van Heel et al., 2018; Blin et al., 2021).^9^ BGCs that shared less than 70% amino acid identity with known clusters were considered as novel (Petrillo et al., 2021).

### Biocontrol evaluation of strain E against basal rot and gray mold of lily

The seedlings of *Lilium* ‘siberia’ from scale cutting propagation were used to evaluate the control effect of strain E on lily base rot. The seedlings were planted in plastic pots (15 cm diameter, 15 cm height) containing peat moss at 25°C/20°C over a 16/8 h light/dark period. The pathogen *F. oxysporum* (accession number: OP216527) was isolated from lily plants with typical symptoms of lily basal rot and was conserved in the Lily Breeding Laboratory, College of Landscape Architecture, Beijing University of Agriculture (Li et al., 2016). *Fusarium oxysporum* spores were prepared according to the aforementioned method, and the spore suspension was adjusted to 1 × 10^6^ spores/ml by diluting with sterile water. The seedlings of *Lilium* ‘siberia’ with a bulb diameter of about 1.5 cm were used for *F. oxysporum* and isolated strains inoculation. For each treatment, nine pots with one seedling in each pot were used for inoculation. After adding 40 ml of bacteria suspension (OD600 = 1) of strain E to the plant root for 3 days, each seedling was inoculated with 40 ml of suspension of *F. oxysporum* spore. The seedlings were inoculated with sterile water as control group. The symptoms of lily basal rot were recorded 6 weeks after inoculation. Disease severity was scored on a scale of 0–5 according to Shahin’s method (Shahin et al., 2011) with slight modifications: 0, health; 1, slight decay; 2, moderate decay; 3, severe decay; 4, very severe decay; 5, completely rotten. Disease incidence was calculated according to Ding’s method (Ding et al., 2021). Disease index (DI) was calculated using the following formula:


$$\mathrm{DI}=\frac{\sum \left(N\times \mathrm{DS}\right)}{T\times 5}\times 100,$$

where *N* is the number of plants at each scale, DS is the disease scale, and *T* is the total number of plants. The relative biocontrol efficacy of the bacterial strains was calculated according to the following equation (Purkayastha et al., 2018):


$$\begin{array}{l} \mathrm{Biocontrol efficacy}\\ =\frac{\mathrm{DI}\;\mathrm{in untreated control}–\mathrm{DI}\;\mathrm{in treatment}}{\mathrm{DI}\;\mathrm{in untreated control}}\times 100\% \end{array}$$

To evaluate the ability of strain E to control gray mold in lily, leaves were excised from *Lilium* ‘siberia’ at the mature flowering stage and disinfected for 30 s in 75% (*v*/*v*) alcohol, followed by rinsing with sterile water thrice. Sterilized leaf segments were placed in Petri dishes with sterile gauze for moisturization. Five microliters of suspension of strain E (OD600 = 1) containing 0.03% Tween 80 were dropped to near the center of the leaf segment. Sterile water containing 0.03% Tween 80 was used as a negative control. The pathogen *Botrytis cinerea* (accession number: OP216528) was isolated from lily plants with typical symptoms of gray mold and conserved in the Lily Breeding Laboratory. Mycelium plugs (0.5 cm in diameter) of *Botrytis cinerea* excised from the actively growing edge of the colonies were inoculated onto the center of the leaf segments. Each treatment consisted of three replicates, and each replicate consisted of three leaf segments per plate. Inoculated leaf segments were incubated at 25°C/20°C (16 h/8 h, light/dark). Lesion diameter (LD) at the site of inoculation was measured using the average *X* and *Y* lengths after 4 days of incubation. The relative biocontrol efficacy of strain E was calculated using the following equation:


$$\begin{array}{l} \mathrm{Biocontrol efficacy}\\ =\frac{\mathrm{LD}\;\mathrm{in untreated control}–\mathrm{LD}\;\mathrm{in treatment}}{\mathrm{LD}\;\mathrm{in untreated control}}\times 100\%. \end{array}$$

### Promoting effect of strain E on the occurrence of scale bulblets

Suspensions of strain E were prepared according to the above method and adjusted to 1 × 10^6^ CFU/ml, 1 × 10^7^ CFU/ml, and 1 × 10^8^ CFU/ml using sterile water. Lily scales were disinfected in 2% sodium hypochlorite solution for 5 min, followed by rinsing with sterile water thrice. Sterilized lily scales were planted in sterilized peat, which was inoculated with 200 ml bacterial suspension per kg of peat. Sterile water was used as a negative control. After 7 weeks of growth at 22°C in the dark, the diameter, root length, and weight of scale bulblets were recorded. Each treatment consisted of three replicates, and each replicate consisted of three lily scales in one pot.

### Construction of GFP-labeled strain E and colonization assay

The plasmid pGFP78, an *Escherichia coli*–*Bacillus subtilis* shuttle vector containing the *GFP* gene controlled by the GFP78 promoter, was electroporated into *B. velezensis* strain E, according to a previously reported method (Zhang et al., 2017; Ding et al., 2021). GFP-labeled *B. velezensis* strain E was selected on LB agar medium supplemented with tetracycline (10 μg/ml) and examined using a fluorescence microscope (Zeiss Axio Scope.A1, Carl Zeiss, Jena, Germany).

Suspensions of GFP-labeled strain E (1 × 10 ^9^ CFU/ml) were prepared according to the aforementioned method. The *Lilium* ‘Siberia’ bulbs were soaked in the suspension of GFP-labeled strain E for 60 min. Sterile water was used as control. Each treatment consisted of three replicates, and each replicate consisted of three plantlets. The root colonization of strain E was detected using previously reported methods with minor modifications (Thomas, 2004; Valdameri et al., 2015; Zhang et al., 2022). To evaluate the epiphytic colonization ability of strain E, 1 g of the lily root from plantlets was collected, cleaned with sterilized water to remove the peat on the root surface, and then agitated in a vortex for 1 min in 5 ml of sterilized water containing 0.03% Tween 80 seven days after inoculation. To evaluate the endophytic colonization ability of strain E on lily root, 1 g of root was disinfected by washing for 30 s in 75% ethanol solution and 3 min in 5% NaClO solution, rinsed three times with sterilized water, and then ground in a sterile mortar and suspended in 9 ml of sterile water. The bacterial suspension obtained was serially diluted and plated on LB agar plates containing tetracycline (10 μg/ml). After incubation overnight at 28°C, the number of bacteria (CFUs) was counted and recorded.

*Lilium* ‘siberia’ seedling planted for 2 months were used to evaluate the ability of leaf colonization of strains E. The leaves of lily seedlings were sprayed with bacterial suspension (1 × 10 ^9^ CFU/ml) of GFP-labeled strain E with 0.03% Tween 80. Sterile water was used as control. Each treatment consisted of three replicates, and each replicate consisted of three seedlings. The leaves were surface washed with sterilized water and dried on sterilized filter paper 7 days after inoculation. Then, 1 g of leaf from each seedling was ground in a sterile mortar and suspended in 9 ml of sterile water. The suspension was diluted tenfold with sterile water and plated on LB agar plates with tetracycline (10 μg/ml), followed by overnight incubation at 28°C for colony count (CFUs).

### Statistical analysis

The data obtained in the study were analyzed using the statistical software SPSS 19.0 (SPSS Inc., Chicago, IL, United States). Duncan’s multiple range test was performed to determine significant differences among the mean values (*p* < 0.05).

## Results

### Isolation and identification of antagonistic bacteria against *Fusarium oxysporum*

**Figure 1:**
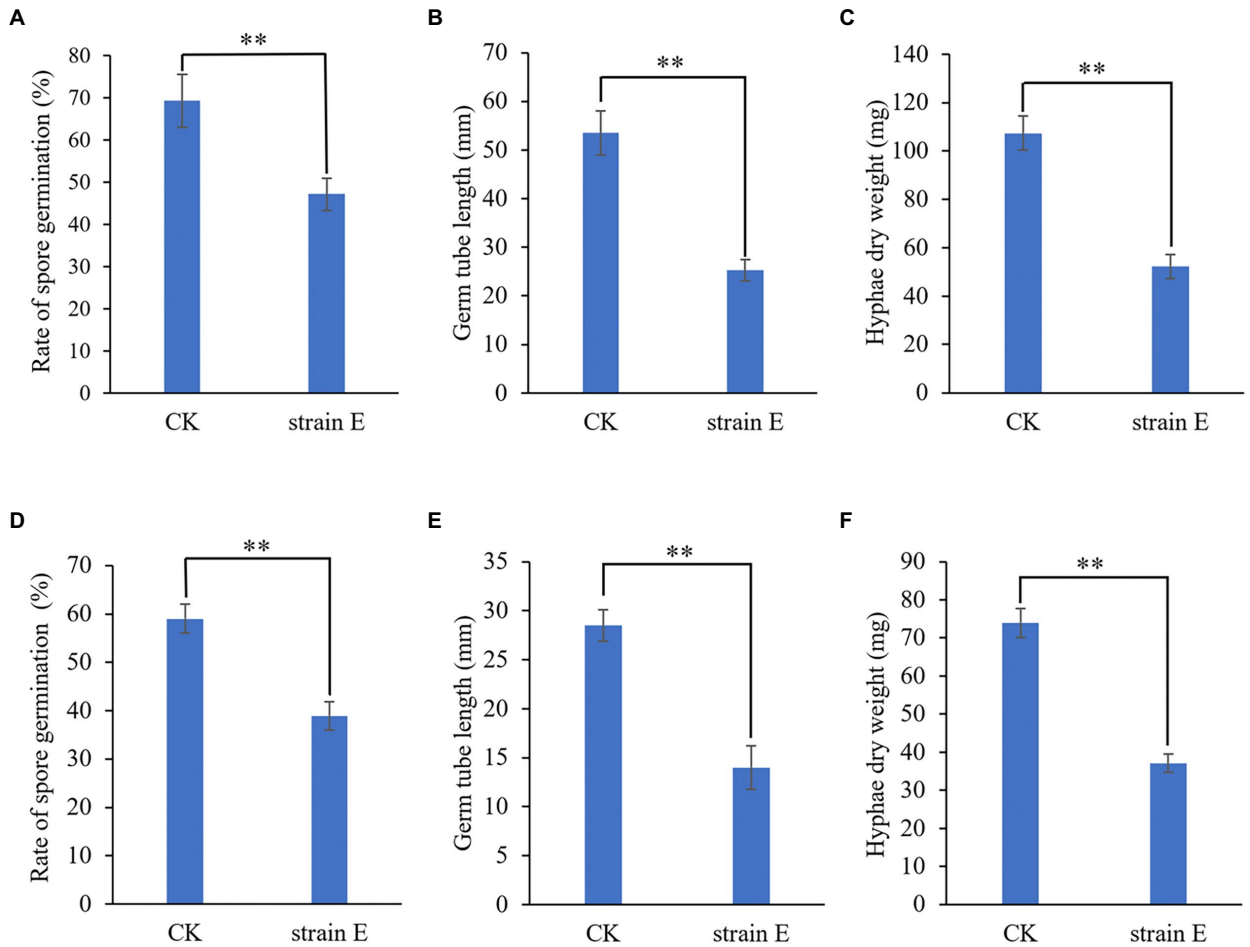
Growth inhibition of *Fusarium oxysporum* and *Botrytis cinerea* by cell-free supernatants of *B. verezensis* strain E. **(A–C)** *Fusarium oxysporum*. **(D–F)** *Botrytis cinerea*. **(A**,**D)** Rate of spore germination. **(C**,**E)** Germ tube length. **(D**,**F)** Hyphae dry weight. LB broth was added to the spore suspension of the fungal pathogen as a control. Asterisks indicate significant difference according to Duncan’s multiple range test. (^**^p < 0.05).

**Figure 2:**
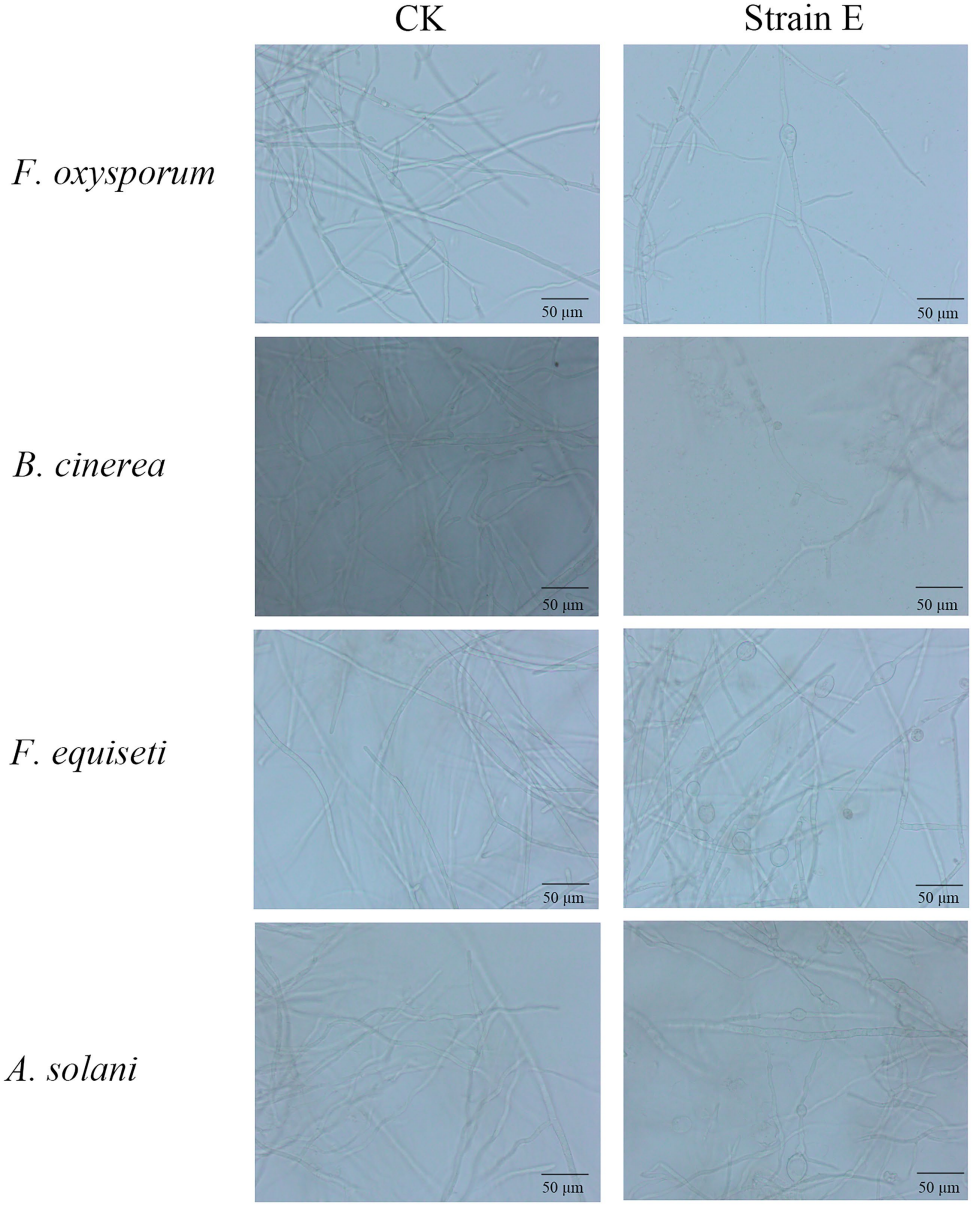
Morphological changes observed in hyphae of fungal pathogens after treatment with strain E. LB broth was used as a control.

Seven bacterial isolates with antagonistic activity against *F. oxysporum* were obtained. The mycelial growth inhibition rate of these bacterial isolates ranged from 42.7 to 86.0% using dual culture assay. Among these, strain E exhibited the highest antifungal activity against *F. oxysporum* (Table 1). The cell-free supernatants of strain E inhibited spore germination, germ tube length and hyphal dry weight of *F. oxysporum* (Figures 1A–C), and caused swelling of hyphae (Figure 2). Strain E was classified as *Bacillus verezensis* based on the BLASTn analysis of the 16S rRNA gene sequence. It shared more than 99% identity with several *Bacillus verezensis* strains.

**Table 1:** *In vitro* antagonistic activity of bacterial strains isolated from soil against *Fusarium oxysporum.*

Strains	Average colony diameter (mm)^*^	Inhibition rate (%)
CK^†^	83.8 ± 0.6a	–
C	49.7 ± 0.5b	42.7 ± 0.6f
D	48.3 ± 0.8c	44.4 ± 1.0e
E	15.2 ± 0.5 g	86.0 ± 0.6a
G	18.0 ± 0.4f	82.4 ± 0.5b
8	19.5 ± 0.2e	80.5 ± 0.3c
11	22.7 ± 0.8d	76.6 ± 1.0d
Z1	48.7 ± 0.1c	43.9 ± 0.1e

* Different letters indicate significant differences (*p* < 0.05) according to Duncan’s multiple range test.

† *Fusarium oxysporum* cultured on PDA plates was used as control.

### Antagonistic activity of isolated bacteria against other plant pathogenic fungi and bacteria

**Figure 3:**
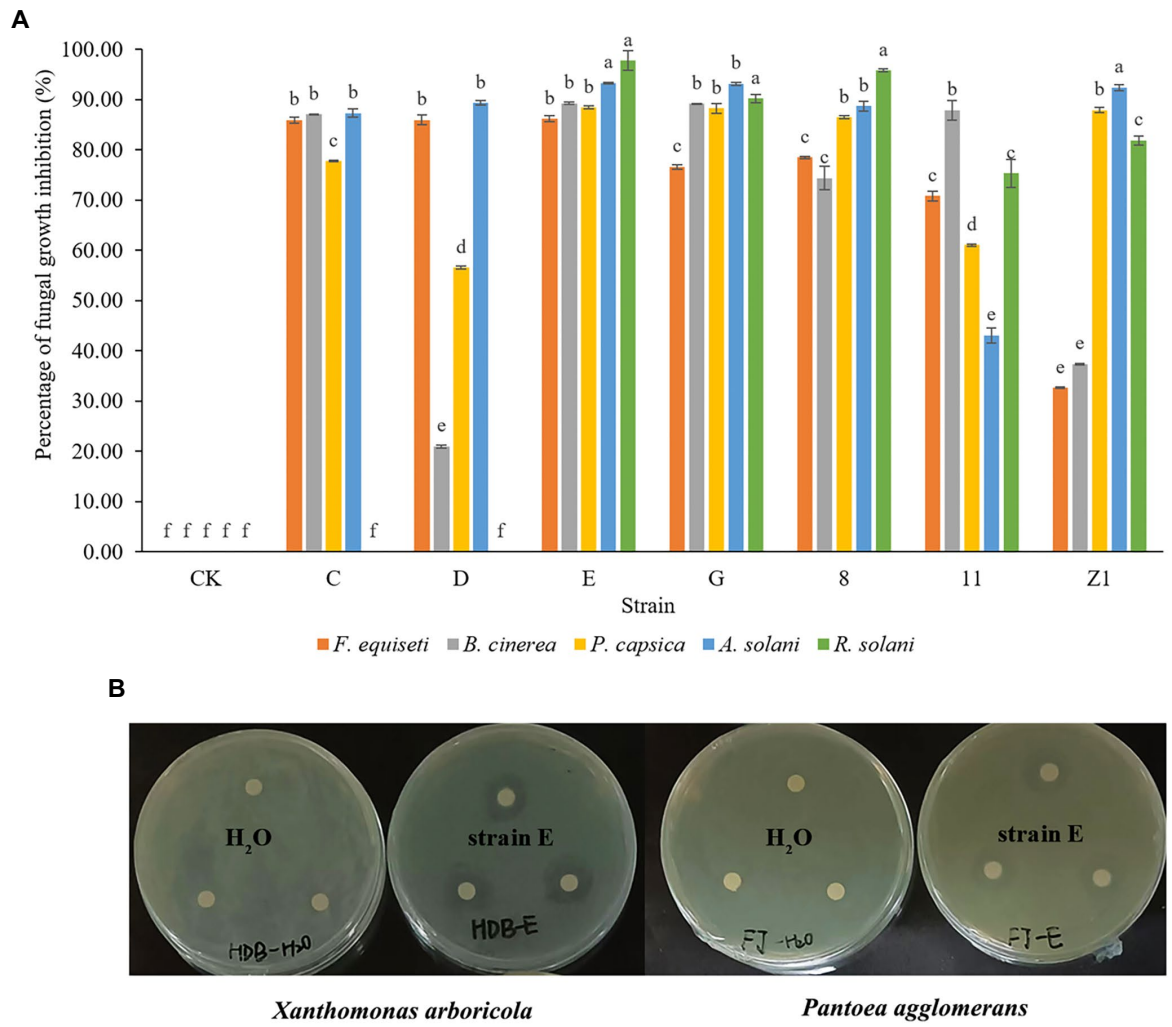
Antagonistic activities of isolated bacterial strains against economically important fungal and bacterial pathogens. **(A)** Antagonistic activity of seven bacterial strains against five fungal pathogens using dual culture assay. Three replicates of each treatment were used. Fungal pathogens cultured on PDA plates was used as control. Bars labeled with different letters indicate significant difference (*p* < 0.05) according to Duncan’s multiple range test. **(B)** Antagonistic activity of strain E against two bacterial pathogens using disc diffusion assay. Sterile water was used as a control.

Using a dual culture assay, all seven bacterial strains tested against *F. oxysporum* were found to display antifungal activity against five economically important fungal pathogens of landscape plants. Among them, *B. verezensis* E had the widest spectrum of antifungal activity and exhibited a good inhibitory effect on *Fusarium equiseti*, *Botrytis cinerea*, *Rizoctonia solani*, *Alternaria solani*, and *Phytophthora capsica*, with percentage of growth inhibition ranging from 86.1 to 97.7% (Figure 3A). The phenomena of death, winding, curling, knotting or swelling of the hyphe of *F. equiseti*, *B. cinerea* and *A. solani* were observed after treatment with cell-free supernatants of strain E (Figure 2). The cell-free supernatants of strain E inhibited spore germination, germ tube length, and hyphal dry weight of *B. cinerea* (Figures 1D–F). The disc diffusion method was used to investigate the *in vitro* antagonistic effects of strain E on two economically important bacterial pathogens, *Xanthomonas arboricola*, and *Pantoea agglomerans* (Yang et al., 2011; Lamichhane, 2014; Fan et al., 2022). After 24 h of incubation, clear inhibition zones around the colonies of strain E were observed on LB plates coated with *Xanthomonas arboricola* and *Pantoea agglomerans* (Figure 3B).

### Effects of *Bacillus verezensis* E on controlling lily basal rot and gray mold

**Figure 4:**
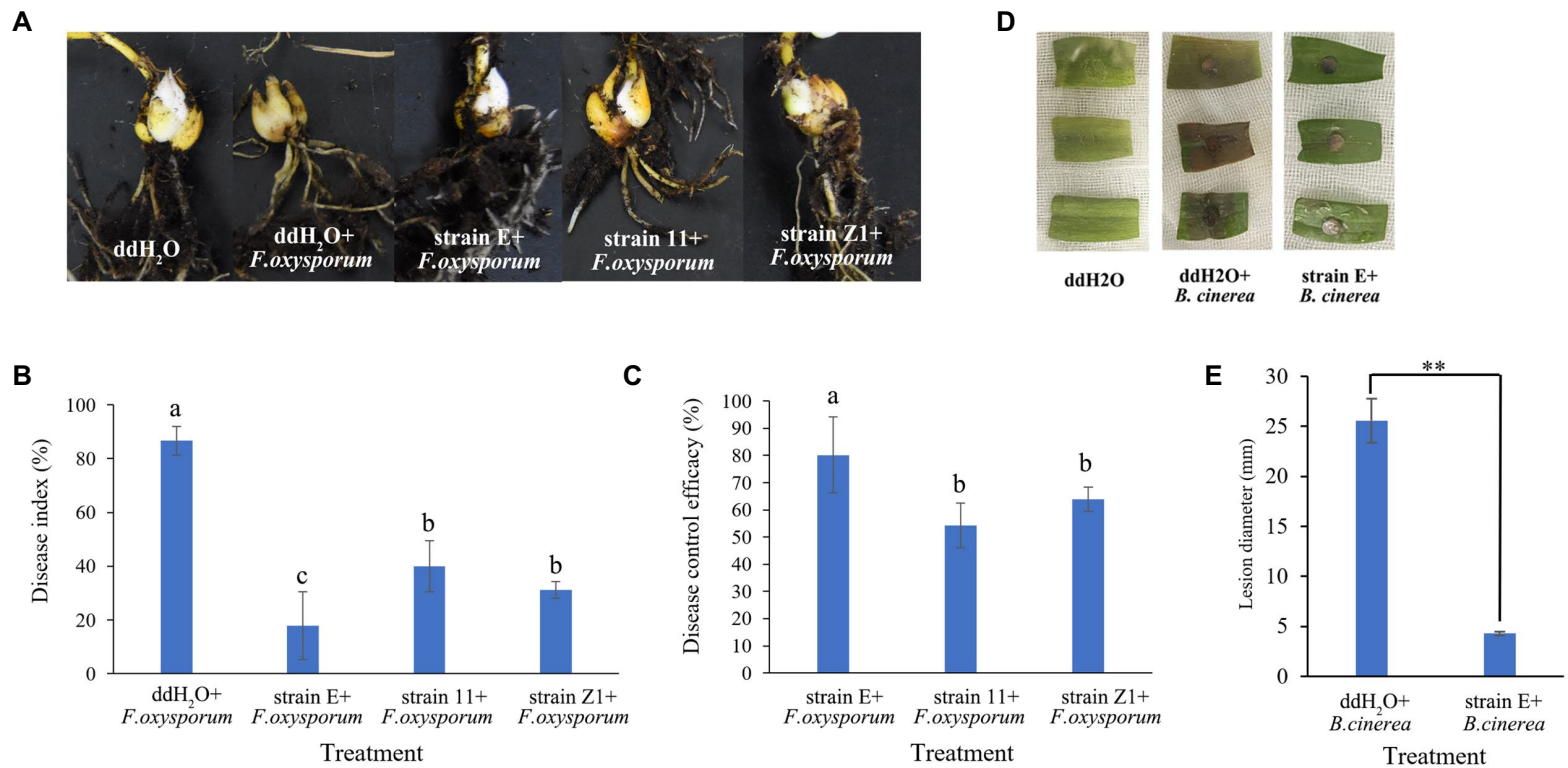
Efficacy of isolated bacterial strains in controlling lily basal rot and gray mold caused by *F. oxysporum* and *B. cinerea, respectively.* **(A)** Lily basal rot. **(B)** Lily gray mold. **(C)** Disease index and **(D)** control efficacy of bacterial strains against lily basal rot. Bars labeled with different letters are significantly different (*p* < 0.05) according to Duncan’s multiple range test. **(E)** Lesion diameter of lily leaf after treatment with ddH_2_O and strain E. Asterisks indicate statistically significant difference according to Duncan’s multiple range test. (^**^p < 0.05).

Three bacterial strains (E, 11, and Z1) with the highest antagonistic activity against *F. oxysporum* were selected to evaluate their ability to control lily basal rot in greenhouses. Strain E exhibited the highest disease control efficacy (80.2%). The disease control efficacies of strains 11 and Z1 were only 54.2 and 64.0%, respectively (Figures 4A–C). The biocontrol activities of *B. verezensis* E against lily gray mold were further evaluated using *in vitro* detached leaf assays. Lily leaves from the control group showed spot symptoms 24 h after inoculation with *B. cinerea*. However, the lily leaves treated with strain E did not show disease symptoms until 48 h after inoculation. The diameter of disease spots in leaves treated with strain E was significantly smaller than that of the control 4 days after inoculation with *B. cinerea*. Therefore, the disease control efficacy of *B. verezensis* strain E could reach 83.0% (Figures 4D,E).

### *Bacillus verezensis* E promotes the occurrence of scale Bulblets

**Figure 5:**
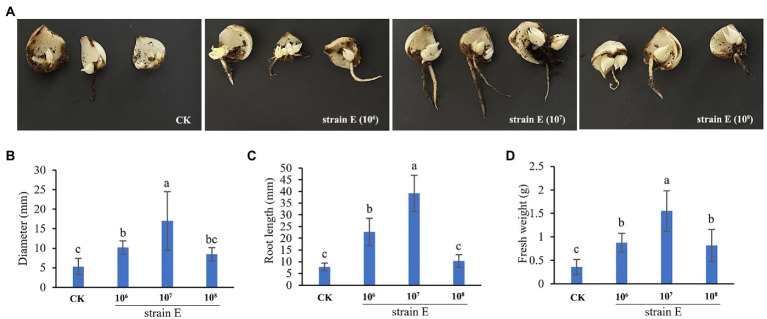
Strain E promotes the occurrence of scale bulblets. **(A)** The occurrence of scale bulblets after treatment with 1 × 10^6^ CFU/ml, 1 × 10^7^ CFU/ml, and 1 × 10^8^ CFU/ml bacterial suspension. **(B)** Diameter of scale bulblets. **(C)** Root length of scale bulblets. **(D)** Fresh weight of scale bulblets. Sterile water was used as a control. Bars labeled with different letters are significantly different (*p* < 0.05) according to Duncan’s multiple range test.

After the lily scales were treated with a bacterial solution of strain E for 6 weeks, the diameter, root length, and fresh weight of scale bulblets in the treatment groups were larger than those in the control group (Figures 5A–D). Among the scales treated with 1 × 10^7^ CFU/ml bacterial suspension product, the scale bulblets had the heaviest fresh weight, largest diameter, and longest root (Figures 5B–D).

### Evaluation of biocontrol activities and plant growth stimulating traits of *Bacillus verezensis* E *in vitro*

**Figure 6:**
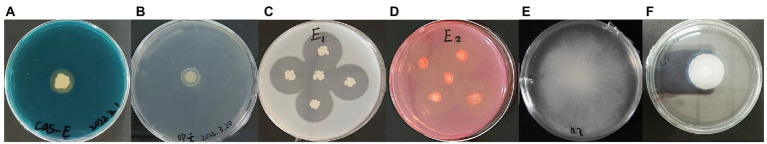
Plant growth promoting and environmental fitting traits of strain E. **(A)** Siderophore production. **(B)** Organic phosphate solubilization. **(C)** Protease production. **(D)** Cellulase production. **(E)** Swimming motility. **(F)** Swarming motility. All assays were performed in three or five repetitions in three independent experiments.

**Figure 7:**
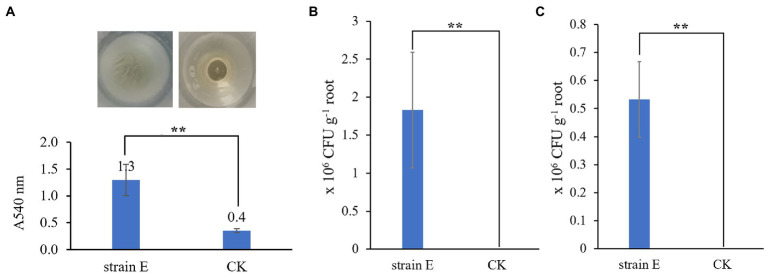
Assays of colonization and biofilm formation of strain E **(A)** Evaluation of the biofilm-forming capacity by crystal violet staining. LB broth was used as a control. Epiphytic **(B)** and endophytic **(C)** colonization of GFP-labeled strain E on the lily root. Sterile water was used as a control. Asterisks indicate statistically significant differences according to Duncan’s multiple range test. (^**^p < 0.05).

Some traits related to biocontrol and plant growth were also detected in strain E. It produced siderophores, proteases and cellulases (Figures 6A,C,D). A very slight halo zone around the bacterial colony in the organic phosphorus solid medium showed that strain E could slightly solubilize organic phosphate (Figure 6B), but not inorganic phosphate (data not shown). The strain E displayed excellent swimming motility, and the plate was almost fully covered by the swimming cells after incubation at 30°C for 11 h (Figure 6E). It also exhibited swarming motility, showing a swarming zone of 3.5 cm diameter after incubation at 30°C for 11 h (Figure 6F), and could completely cover the plate in 33 h. In addition, the crystal violet staining assay showed that strain E formed biofilms in polypropylene tube (Figure 7A).

### Colonization of *Bacillus verezensis* E on lily root and leaf

To investigate root and leaf colonization of *B. velezensis* strain E, a GFP-labeled strain E was constructed. GFP-labeled strain E exhibited strong colonization activity on the lily root. For epiphytic colonization, the GFP-labeled strain E (1.8 × 10^6^ CFU per gram root) was isolated on the root surface at 7 days after inoculation (Figure 7B). For endophytic colonization, the density of GFP-labeled strain E was 0.5 × 10^6^ CFU per gram of surface-disinfected root (Figure 7C). However, no colonies of GFP-labeled strain E were detected from the lily leaves using our method (data not shown). Therefore, the density of GFP-labeled strain E was less than 100 CFU per gram of leaf after inoculation for 7 days.

### Sequencing and analysis of *Bacillus verezensis* E genome

**Figure 8:**
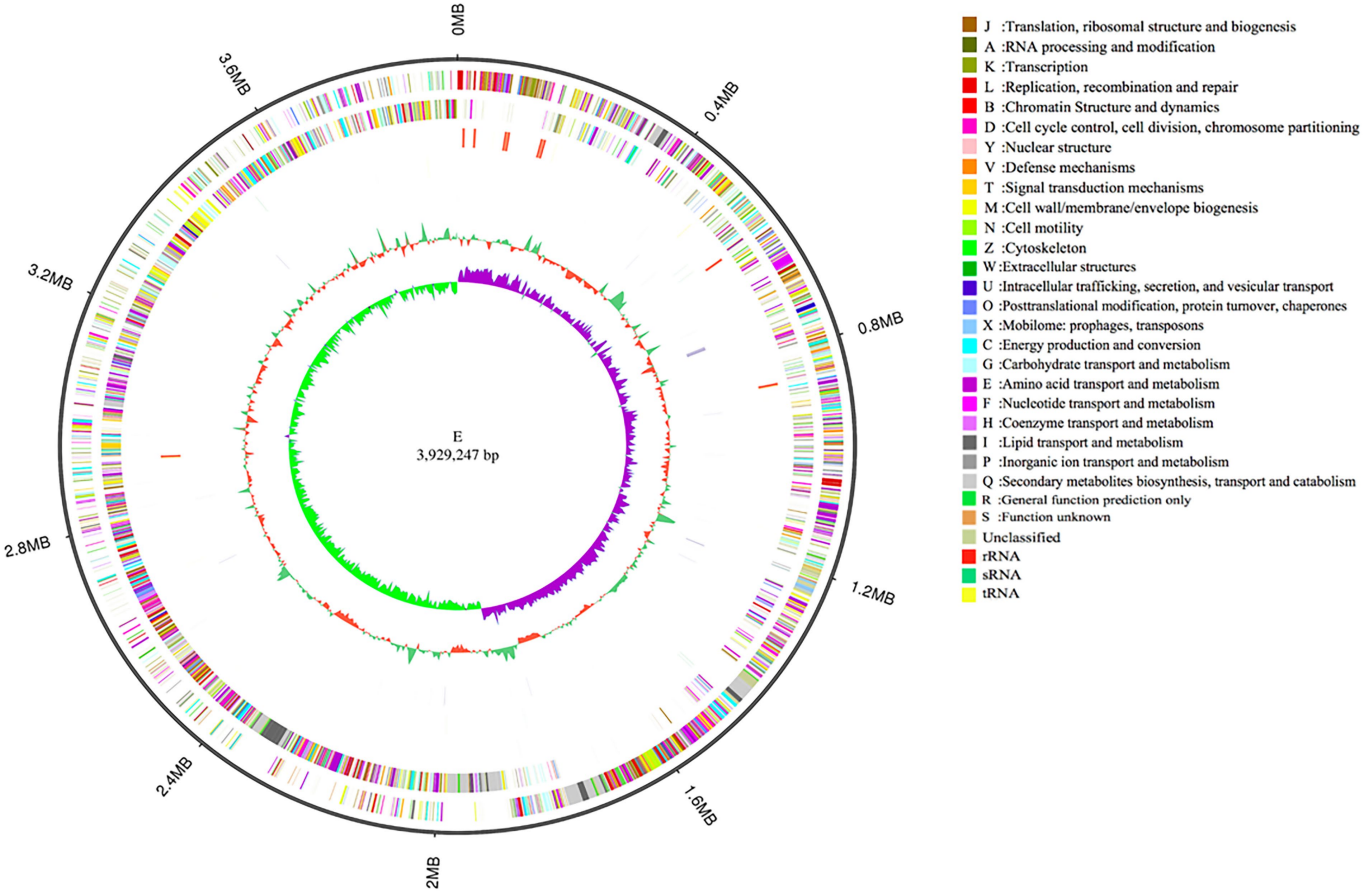
Genomic completion map of strain E. The size and scale of genome is shown in the outermost circle. Outer to inner rings represent forward and reverse strand genes annotated according to COG, forward and reverse strand ncRNA, repeat, GC content and GC-SKEW.

To investigate the biocontrol mechanisms of *B. velezensis* strain E, its complete genome was sequenced and analyzed. The genome sequence of strain E consisted of a complete circular chromosome (Figure 8), which was 3,929,247 bp in size, and had a GC content of 47.32%. No plasmid was detected in strain E. The whole genome of strain E contained 4,056 protein-coding genes (88.76% of the genome length), 27 tRNA, 86 rRNA genes, and 272 repeat elements. The total length of protein-coding genes was 3,487,731 bp with an average length of 859.89 bp (Table 2).

**Table 2:** Statistics of strain E genome assembly.

Items	Results
Total length (bp)	3,929,247
GC content (%)	47.32
Total number of protein-coding genes	4,056
Total length of protein-coding genes (bp)	3,487,731
Average length of protein-coding genes (bp)	859.89
Number of rRNA genes	86
Number of tRNA genes	27
Number of sRNA	31
Repeats	272

**Figure 9:**
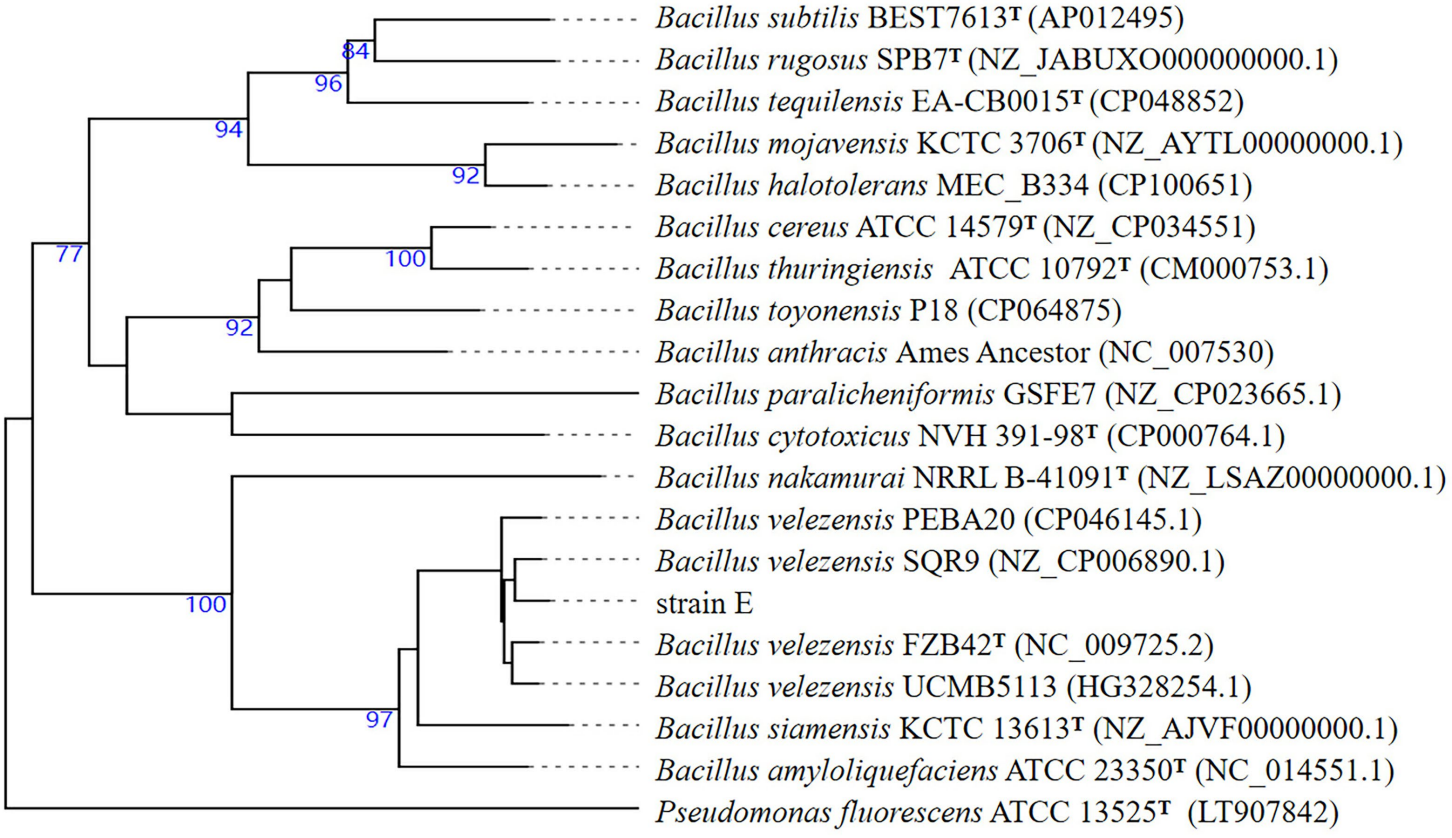
Sequence-based phylogenomic tree of strain E constructed on TYGS. Type strains are indicated by ^T^. Reference sequences were obtained from the GenBank database. Bootstrapping was performed using 1,000 replicates.

Previously, strain E was identified as *B. velezensis* based on 16S rDNA gene sequences. To further verify this classification, the whole genome sequence of strain E was compared with those of other *Bacillus* strains using the Type (strain) Genome Server. Strain E was found to belong to *B. velezensis* (Figure 9). Strain E was further compared with other closely related *Bacillus* strains at the genome level using ANI and dDDH analyses. The OrthoANI (98.73, 98.22, 98.56, and 98.20%) and dDDH (89.0, 84.7, 84.6, and 84.3%) values between strain E and four *Bacillus velezensis* strains were higher than the published species thresholds (95–96% for OrthoANI, 70% for dDDH; Table 3; Meier-Kolthoff et al., 2013; Yoon et al., 2017). Because the analysis results from the ANI and dDDH methods were consistent with those from the genome-based phylogenetic analysis, strain E was classified as *B. velezensis*.

**Table 3:** Pairwise dDDH and orthoANI values between strain E and other *Bacillus* strains.

Bacterial strains	OrthoANI (%)	dDDH (%)
*Bacillus velezensis* SQR9	98.73	89.0
*Bacillus velezensis* FZB42^T^	98.22	84.7
*Bacillus velezensis* UCMB5113	98.56	84.6
*Bacillus velezensis* PEBA20	98.20	84.3
*Bacillus siamensis* KCTC 13613^T^	94.37	56.3
*Bacillus amyloliquefaciens* ATCC23350^T^	94.01	55.1
*Bacillus nakamurai* NRRL B-41091^T^	86.30	30.7

Type strains are indicated by ^T^.

### Analysis of biosynthetic gene clusters of secondary metabolites in *Bacillus verezensis* E

To better understand this antagonistic activity, BGCs of secondary metabolites were predicted and located in the genome of strain E using antiSMASH. Twelve putative BGCs with potential antimicrobial activities were identified. Among them, seven BGCs encoding synthetases of surfactin, macrolactin H, bacillaene, fengycin, difficidin, bacillibactin and bacilysin were highly homologous to those of known compounds in the MiBIG database and had similar gene structures to other *B. velezensis* strains (Table 4; Supplementary Figure 1). The remaining five BGCs involved in the biosynthesis of terpenes, PKS-like, T3PKS, and lanthipeptide-class-ii did not match the clusters of known compounds in the MiBIG database (Table 4). However, they were common in other *B. velezensis* strains based on ClusterBlast analysis of antiSMASH, with more than 95% gene similarity (Supplementary Figure 1).

**Table 4:** Analysis of secondary metabolite biosynthetic gene clusters (BGCs) in strain E.

Region	Synthetase type	Predicted size (bp)	Most similar known cluster	Similarity (%)	MIBiG accession
1	NRPS	63,975	Surfactin NRP:Lipopeptide	82	BGC0000433
2	PKS-like	41,245	–	–	–
3	terpene	17,405	–	–	–
4	lanthipeptide-class-ii	28,889	–	–	–
5	transAT-PKS	87,825	Macrolactin H Polyketide	100	BGC0000181
6	transAT-PKS,T3PKS,NRPS	100,552	Bacillaene Polyketide+NRP	100	BGC0001089
7	NRPS,transAT-PKS,betalactone	134,285	Fengycin NRP	100	BGC0001095
8	terpene	20,549	–	–	–
9	T3PKS	41,101	–	–	–
10	transAT-PKS	93,781	Difficidin Polyketide+NRP	100	BGC0000176
11	NRPS,RiPP-like	51,790	Bacillibactin NRP	100	BGC0000309
12	other	41,419	Bacilysin Other	100	BGC0001184

Genome analysis was performed using antibiotic and secondary metabolite analysis shell (antiSMASH) and the MIBiG database. PKS, polyketide synthetase; NRPS, non-ribosomal peptide synthetase; RiPP, post-translationally modified peptide.

To detect potential novel bacteriocin BGCs, the genome sequence of *B. velezensis* strain E was mined using the BAGEL4. Three areas of interest (AOI) were predicted to produce potential bacteriocins (Supplementary Figure 2). Among them, bacteriocin BGCs from AOI_01 and AOI_03 corresponded to those of regions11 and region 4 from antiSMASH analysis, respectively. AOI_02, encoding LCI, was identified using BAGEL4 instead of antiSMASH. One product of AOI_03, which shared ≤ 70% of similarity with known sequences in BAGEL4 database, were classified as novel bacteriocin (Supplementary Figure 2).

### Analysis of *Bacillus verezensis* E genes involved in colonization, plant growth, and biological control

The genes involved in biocontrol, plant growth, and colonization were identified in strain E (Supplementary Table 1). To exert beneficial effects on plants, bacteria must colonize the plants. Many studies have demonstrated that bacterial colonization depends on their motility, chemotaxis, and biofilm formation abilities (Beauregard et al., 2013; Allard-Massicotte et al., 2016; Xu et al., 2019). A set of genes involved in biofilm matrix formation (*epsA-O*, *tapA-sipW-tasA*, and *bslA*) and regulation (*spo0A*, *sinI*, *sinR*, *ylbF*, *ymcA*, and *yaaT*) were identified from the genome of strain E. Bacteria must adhere to plant surfaces before biofilm formation (Xu et al., 2019). The genome of strain E contained a complete set of genes implicated in chemotaxis and motility, including *cheR*, *cheV*, *hfq*, *motA*, *motB*, *swrA*, and *swrB*, six genes encoding methyl-accepting chemotaxis proteins, and 30 genes in the *fla/che* operon (EGL001674-EGL001705). Beneficial bacteria can promote plant growth and development by improving the solubilization of mineral nutrients and synthesizing specific compounds to promote plant growth (Bhattacharyya and Jha, 2012; Aeron et al., 2020; Samaras et al., 2021). Multiple genes associated with plant growth-promoting activity in strain E were predicted, including the production of indole-3-acetic acid (IAA)(*trpABFCDE* operon, *dhaS*, and *ysnE*), siderophores (*dhbF*, *dhbB*, *dhbE*, *entC*, and *entA*), organic phosphorus mineralization (*phoA*, *phoB*, *phoD*, *phoR*, *phoP*, and *pstB*), and nitrogen metabolism (*nos*, *narGHJI* operon, *nasA, nirD*, *nirB*, and *nirK*).

## Discussion

Lily basal rot and lily gray mold are important diseases that endanger lily production (Cui et al., 2018; He et al., 2019). Although biological control has been widely used to control many plant diseases, the biological control of lily diseases has not been extensively studied. Previously, one endophytic fungus, *Acremonium* sp., and three endophytic bacteria, *Bacillus velezensis* Lle-9, *Bacillus stratosphericus* LW-03, and *Paenibacillus polymyxa* SK1, were isolated from *Lilium davidii*, *Lilium leucanthum*, *Lilium wardii*, and *Lilium lancifolium*, respectively, all of which showed antifungal effects against *Fusarium oxysporum*, *Botrytis cinerea*, *Botryosphaeria dothidea*, and *Fusarium fujikuroi in vitro* (Khan et al., 2020a,b,c, 2021). These four microorganisms promoted lily growth, including increases in plant height, leaf length, and root length, which were significantly higher than those of the non-inoculated control plants. However, the effects of these four antagonistic microorganisms on diseases affecting lily have not been studied. In the present study, a new biocontrol, *B. velezensis* strain E, with broad-spectrum antimicrobial activity against filamentous fungi and Gram-negative bacteria, was isolated from a continuous lily cropping field. Strain E could effectively control gray mold and basal rot of lily in greenhouse experiments and *in vitro* detached leaf assays, respectively. The scale cutting propagation of lily is a low-cost and easy operation; however, it has a low propagation coefficient and bulblet formation is slow (Sun et al., 2009). In this study, *B. velezensis* strain E could effectively promote *in vitro* regeneration of bulblets by scale cutting, which is an important way to produce lily bulbs. Hence, *B. velezensis* strain E could potentially be used not only as a BCA for lily to control basal rot and gray mold disease, but also as a microbial fertilizer in lily production.

*Bacillus* species, which are widely distributed in the environment, have been extensively studied, because they can produce biologically active secondary metabolites that inhibit pathogen growth and promote plant growth (Zahir et al., 2003; Rabbee et al., 2019). *Bacillus velezensis*, a latetype isoform of *B. amyloliquefaciens*, is a new type of biocontrol bacterium (Dunlap et al., 2016; Yan et al., 2021). *Bacillus velezensis* has wide-spectrum biological activities against various plant pathogens, such as *Phytophthora infestans*, *Pectobacterium carotovorum*, *Glomerella glycines*, *Rhizoctonia solani*, *Alternaria alternate*, and *Blumeria graminis* (Cai et al., 2017; Saravanan et al., 2021; Yan et al., 2021; Htwe Maung et al., 2022). However, there is no report of the antagonistic effect of *B. velezensis* on *Fusarium equiseti*, *Xanthomonas arboricola*, and *Pantoea agglomerans*. Among them, *Pantoea agglomerans* can cause bacterial shot-hole disease of plum, bacterial soft rot of *Dioscorea opposita*, blight disease on *Solanum muricatum* and oat, necrotic disease on *Ziziphus jujuba*, brown apical necrosis of walnut, and bacterial shot-hole disease of plum (Yang et al., 2011; She et al., 2019; Moloto et al., 2020; She et al., 2021; Fan et al., 2022; Wang et al., 2022). *Xanthomonas arboricola* can cause bacterial canker and spot and bacterial blight diseases of stone fruit, almond, and walnut trees, respectively (Lamichhane, 2014). Both of them are economically important bacterial pathogens and cause huge economic losses. In this study, *B. velezensis* strain E could effectively antagonize these pathogens. Our results provide a potentially microbial resource for the biocontrol of diseases caused by these pathogens.

To exert beneficial effects on plants, bacteria must colonize the plant. The GFP-labeled *B. velezensis* E could not be clearly distinguished from the roots and leaves of lily using confocal microscopy because of the autofluorescence of lily roots and leaves. Therefore, antibiotics were added to the plates to determine the colonization density of GFP-labeled strain E in the lily roots and leaves. In this study, lily roots could be colonized epiphytically and endophytically by strain E after 7 days of inoculation. This result was consistent with the previous report (Synek et al., 2021). In that study endophytic colonization of *Arabidopsis* root was found to occur by the passive and active entry of a beneficial endophyte *Enterobacter* sp. SA187. And the endophyte SA187 could be attracted to the meristematic zone of growing roots and colonized epiphytically in the elongation zone. Therefore, we believe that strain E is an endophytic bacterium. Although the fermentation suspension of strain E had a good control effect against *Botrytis cinerea* in *in vitro* detached leaf assays, it could not be detected on lily leaves in the colonization experiment. However, the BCF of strain E has a strong destructive effect on *Botrytis cinerea*; therefore, the practical application effect of strain E on controlling lily gray mold must be studied further.

## Data availability statement

The data presented in the study are deposited in the NCBI database, accession number ON764297 (16S rDNA sequence of strain E, https://www.ncbi.nlm.nih.gov/nuccore/ON764297.1/) and CP099460 (whole genome sequence of strain E, https://www.ncbi.nlm.nih.gov/nuccore/CP099460).

## Author contributions

XH planned the experiments and wrote the manuscript. XH, BL, JW, JS, MT, MW, XZ, WS, YM, JM, YQ, YL, and WW performed the experiments. XH, BL, JW, and JS analyzed the data. All authors contributed to the article and approved the submitted version.

## Funding

This research was funded by the Beijing Municipal Education Commission (KM201810020009) and the Research Fund for Academic Degree and Graduate Education of Beijing University of Agriculture (2022YJS037).

## Conflict of interest

The authors declare that the research was conducted in the absence of any commercial or financial relationships that could be construed as a potential conflict of interest.

## Publisher’s note

All claims expressed in this article are solely those of the authors and do not necessarily represent those of their affiliated organizations, or those of the publisher, the editors and the reviewers. Any product that may be evaluated in this article, or claim that may be made by its manufacturer, is not guaranteed or endorsed by the publisher.

## Footnotes

^1^ https://www.broadinstitute.org/gatk/

^2^ http://www.cbcb.umd.edu/software/glimmer/

^3^ http://rfam.xfam.org/

^4^ http://tandem.bu.edu/trf/trf.html

^5^ http://ggdc.dsmz.de/home.php

^6^ http://www.ezbiocloud.net

^7^ https://tygs.dsmz.de

^8^ https://antismash.secondarymetabolites.org

^9^ http://bagel4.molgenrug.nl
