# Supplementary material for: Genomics assisted functional characterization of Bacillus velezensis E as a biocontrol and growth promoting bacterium for lily
Source: Front Microbiol. 2022 Nov 30;13:976918. doi: 10.3389/fmicb.2022.976918 (PMC9748698; doi:10.3389/fmicb.2022.976918)
Supplement: Supplementary file 1 [file Table_1.DOCX]

Supplementary Table 1 Genes involved in plant growth promotion and biocontrol activities in the strain E genome.

| Function | Gene name | Gene Annotation | E. C. Number | Gene ID |
| --- | --- | --- | --- | --- |
| Siderophores production |  |  |  |  |
|  | *dhbF* | nonribosomal peptide synthetase DhbF | -- | EGL003064 |
|  | *dhbF* | nonribosomal peptide synthetase DhbF | -- | EGL003065 |
|  | *dhbB* | bifunctional isochorismate lyase/aryl carrier protein | 3.3.2.1 6.3.2.14 | EGL003066 |
|  | *dhbE* | 2,3-dihydroxybenzoate-AMP ligase | 6.3.2.14 2.7.7.58 | EGL003067 |
|  | *entC* | isochorismate synthase | 5.4.4.2 | EGL003068 |
|  | *entA* | 2,3-dihydro-2,3-dihydroxybenzoate dehydrogenase | 1.3.1.28 | EGL003069 |
| IAA production |  |  |  |  |
|  | *trpA* | Tryptophan synthase alpha chain | 4.2.1.20 | EGL002194 |
|  | *trpB* | Tryptophan synthase beta chain | 4.2.1.20 | EGL002195 |
|  | *trpF* | Phosphoribosylanthranilate isomerase | 5.3.1.24 | EGL002196 |
|  | *trpC* | Indole-3-glycerol phosphate synthase | 4.1.1.48 | EGL002197 |
|  | *trpD* | Anthranilate phosphoribosyltransferase | 2.4.2.18 | EGL002198 |
|  | *trpE* | Anthranilate synthase component I | 4.1.3.27 | EGL002199 |
|  | *dhaS* | Putative aldehyde dehydrogenase DhaS | -- | EGL002014 |
|  | *ysnE* | putative N-acetyltransferase | 2.3.1.- | EGL003764 |
| Nitrogen metabolism |  |  |  |  |
|  | *nos* | nitric-oxide synthase, bacterial | 1.14.14.47 | EGL000753 |
|  | *narI* | nitrate reductase gamma subunit | 1.7.5.1 1.7.99.- | EGL003667 |
|  | *narJ* | nitrate reductase molybdenum cofactor assembly chaperone | -- | EGL003668 |
|  | *narH* | nitrate reductase / nitrite oxidoreductase, beta subunit | 1.7.5.1 1.7.99.- | EGL003669 |
|  | *narG* | nitrate reductase / nitrite oxidoreductase, alpha subunit | 1.7.5.1 1.7.99.- | EGL003670 |
|  | *nirD* | nitrite reductase (NADH) small subunit | 1.7.1.15 | EGL000323 |
|  | *nirB* | nitrite reductase (NADH) large subunit | 1.7.1.15 | EGL000324 |
|  | *nasA* | assimilatory nitrate reductase catalytic subunit | 1.7.99.- | EGL000325 |
|  | *nirB* | nitrite reductase (NADH) large subunit | 1.7.1.15 | EGL000326 |
|  | *narK* | Nitrate/nitrite transporter | -- | EGL000328 |
| Phosphate solubilization |  |  |  |  |
|  | *phoA, phoB* | alkaline phosphatase | 3.1.3.1 | EGL000947 |
|  | *phoD* | alkaline phosphatase D | 3.1.3.1 | EGL000262 |
|  | *phoR* | two-component system, OmpR family, phosphate regulon sensor histidine kinase PhoR | 2.7.13.3 | EGL000378 |
|  | *phoR* | two-component system, OmpR family, phosphate regulon sensor histidine kinase PhoR | 2.7.13.3 | EGL002783 |
|  | *phoP* | two-component system, OmpR family, alkaline phosphatase synthesis response regulator PhoP | -- | EGL002784 |
|  | *pstB* | phosphate transport system ATP-binding protein | 7.3.2.1 | EGL002456 |
| Chemotaxis and motility |  |  |  |  |
|  | *mcpC* | methyl-accepting chemotaxis protein | -- | EGL001428 |
|  | *mcpA* | methyl-accepting chemotaxis protein | -- | EGL002983 |
|  | *mcpB* | methyl-accepting chemotaxis protein | -- | EGL002985 |
|  | *tlpA* | methyl-accepting chemotaxis protein | -- | EGL002984 |
|  | *tlpB* | methyl-accepting chemotaxis protein | -- | EGL002982 |
|  | *hemAT* | methyl-accepting chemotaxis protein | -- | EGL001051 |
|  | *cheR* | chemotaxis protein methyltransferase CheR | 2.1.1.80 | EGL002203 |
|  | *cheV* | two-component system, chemotaxis family, chemotaxis protein CheV | -- | EGL001435 |
|  | *hfq* | host factor-I protein | -- | EGL001798 |
|  | *flgB* | flagellar basal-body rod protein FlgB | -- | EGL001674 |
|  | *flgC* | flagellar basal-body rod protein FlgC | -- | EGL001675 |
|  | *fliE* | flagellar hook-basal body complex protein FliE | -- | EGL001676 |
|  | *fliF* | flagellar M-ring protein FliF | -- | EGL001677 |
|  | *fliG* | flagellar motor switch protein FliG | -- | EGL001678 |
|  | *fliH* | flagellar assembly protein FliH | -- | EGL001679 |
|  | *fliI* | flagellum-specific ATP synthase | 3.6.3.50 | EGL001680 |
|  | *fliJ* | flagellar FliJ protein | -- | EGL001681 |
|  | *fliK* | flagellar hook-length control protein FliK | -- | EGL001684 |
|  | *flgD* | flagellar basal-body rod modification protein FlgD | -- | EGL001685 |
|  | *flgE* | flagellar hook protein FlgE | -- | EGL001686 |
|  | *flbD* | flagellar protein FlbD | -- | EGL001687 |
|  | *fliL* | flagellar FliL protein | -- | EGL001688 |
|  | *fliM* | flagellar motor switch protein FliM | -- | EGL001689 |
|  | *fliNY, fliN* | flagellar motor switch protein FliN/FliY | -- | EGL001690 |
|  | *cheY* | two-component system, chemotaxis family, chemotaxis protein CheY | -- | EGL001691 |
|  | *fliOZ, fliO* | flagellar protein FliO/FliZ | -- | EGL001692 |
|  | *fliP* | flagellar biosynthetic protein FliP | -- | EGL001693 |
|  | *fliQ* | flagellar biosynthetic protein FliQ | -- | EGL001694 |
|  | *fliR* | flagellar biosynthetic protein FliR | -- | EGL001695 |
|  | *flhB* | flagellar biosynthetic protein FlhB | -- | EGL001696 |
|  | *flhA* | flagellar biosynthesis protein FlhA | -- | EGL001697 |
|  | *flhF* | flagellar biosynthesis protein FlhF | -- | EGL001698 |
|  | *flhG, fleN* | flagellar biosynthesis protein FlhG | -- | EGL001699 |
|  | *cheB* | two-component system, chemotaxis family, protein-glutamate methylesterase/glutaminase | 3.1.1.61 3.5.1.44 | EGL001700 |
|  | *cheA* | two-component system, chemotaxis family, sensor kinase CheA | 2.7.13.3 | EGL001701 |
|  | *cheW* | purine-binding chemotaxis protein CheW | -- | EGL001702 |
|  | *cheC* | chemotaxis protein CheC | -- | EGL001703 |
|  | *cheD* | chemotaxis protein CheD | 3.5.1.44 | EGL001704 |
|  | *fliA* | RNA polymerase sigma factor for flagellar operon FliA | -- | EGL001705 |
|  | *motB* | chemotaxis protein MotB | -- | EGL001394 |
|  | *motA* | chemotaxis protein MotA | -- | EGL001395 |
|  | *motA* | chemotaxis protein MotA | -- | EGL002861 |
|  | *motB* | chemotaxis protein MotB | -- | EGL002862 |
|  | *swrA* | Swarming motility protein SwrA | -- | EGL003445 |
|  | *swrB* | Swarming motility protein SwrB | -- | EGL001706 |
| Biofilm formation and regulation |  |  |  |  |
|  | *epsA-O* | Polysaccharide biosynthesis protein | -- | EGL003348- EGL003362 |
|  | *tasA* | spore coat-associated protein | -- | EGL002423 |
|  | *sipW* | signal peptidase I | 3.4.21.89 | EGL002424 |
|  | *tapA* | TasA anchoring/assembly protein | -- | EGL002425 |
|  | *bslA* | biofilm surface layer protein | -- | EGL002972 |
|  | *sinI* | antagonist of SinR | -- | EGL002421 |
|  | *sinR* | HTH-type transcriptional regulator SinR | -- | EGL002422 |
|  | *spo0A* | two-component system, response regulator, stage 0 sporulation protein A | -- | EGL002383 |
|  | *ylbF* | Cell fate regulator YlbF | -- | EGL000989 |
|  | *ymcA* | Cell fate regulator YmcA | -- | EGL001763 |
|  | *yaaT* | Cell fate regulator YaaT | -- | EGL000036 |
