# Supplementary figures and images for: Genomics assisted functional characterization of Bacillus velezensis E as a biocontrol and growth promoting bacterium for lily
Source: Front Microbiol. 2022 Nov 30;13:976918. doi: 10.3389/fmicb.2022.976918 (PMC9748698; doi:10.3389/fmicb.2022.976918)

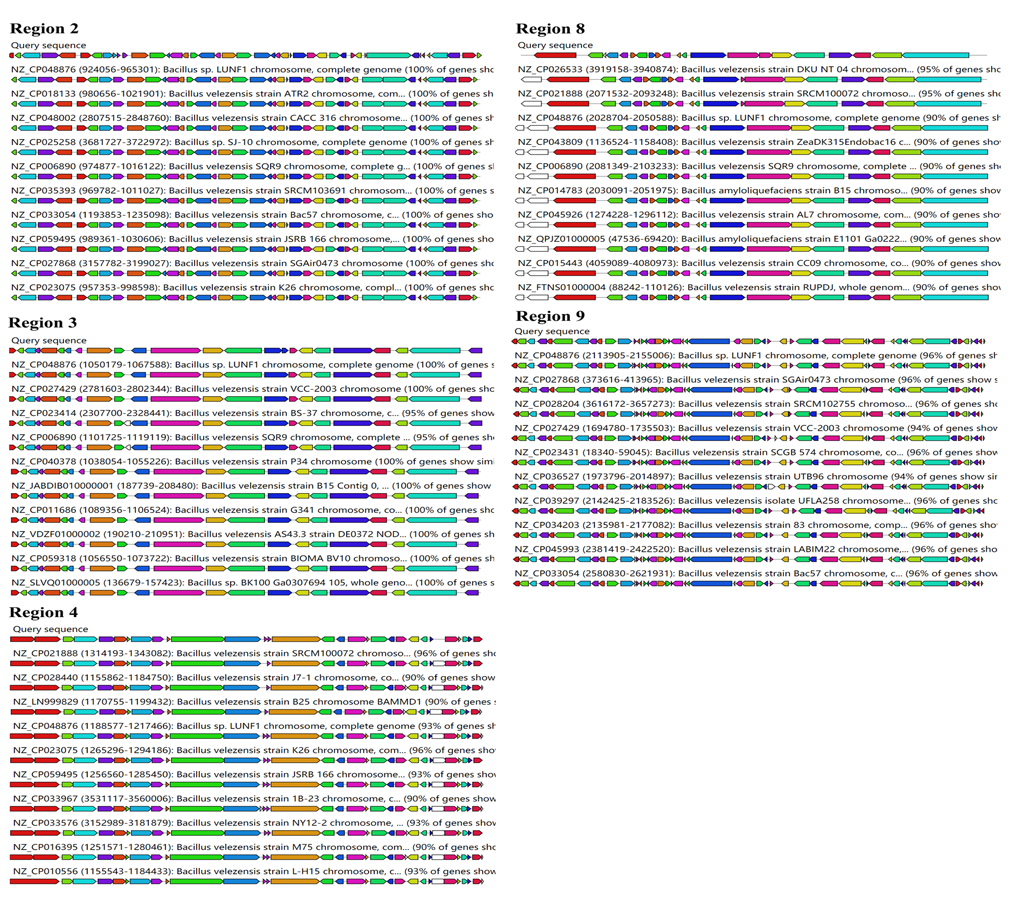

Supplement: Supplementary file 2 [file Image_1.TIF]

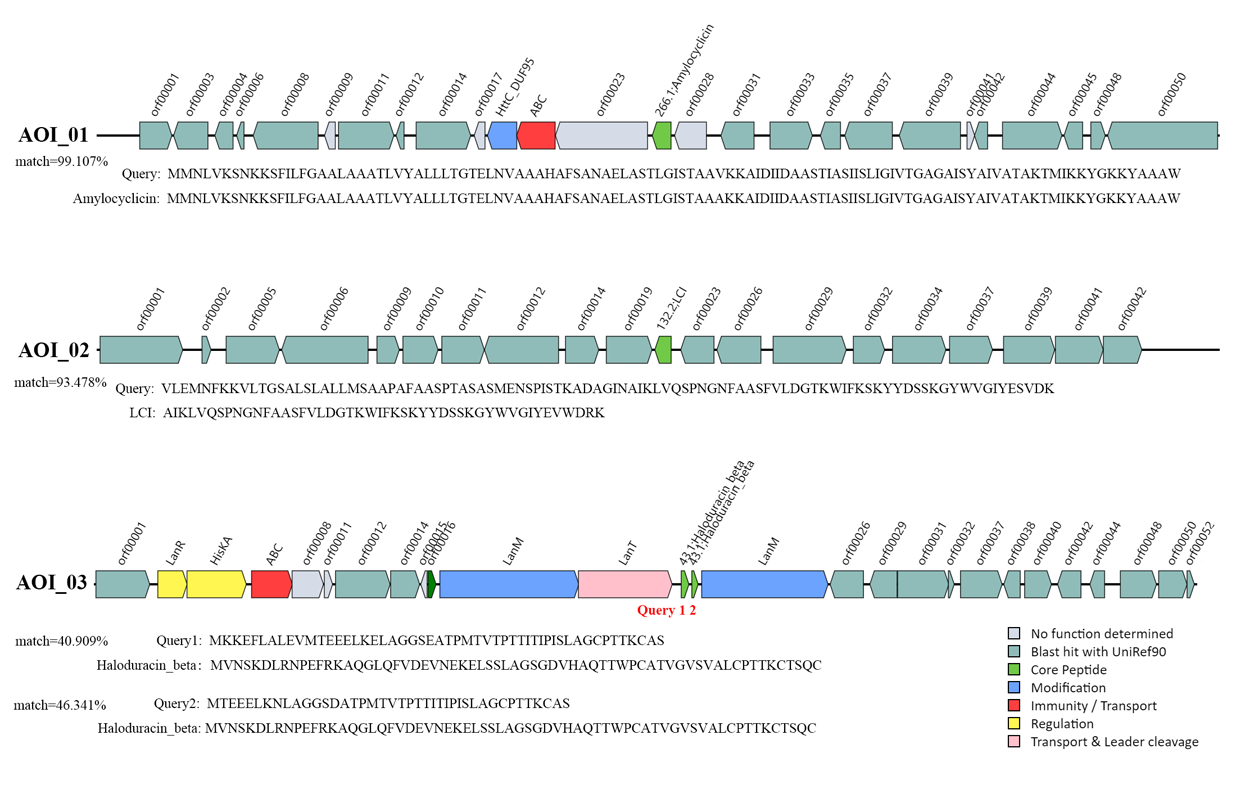

Supplement: Supplementary file 3 [file Image_2.TIF]
